# Unveiling the Role of Side Chain for Improving Nonvolatile Characteristics of Conjugated Polymers‐Based Artificial Synapse

**DOI:** 10.1002/advs.202400304

**Published:** 2024-02-26

**Authors:** Junho Sung, Sein Chung, Yongchan Jang, Hyoik Jang, Jiyeon Kim, Chan Lee, Donghwa Lee, Dongyeong Jeong, Kilwon Cho, Youn Sang Kim, Joonhee Kang, Wonho Lee, Eunho Lee

**Affiliations:** ^1^ Department of Chemical Engineering Kumoh National Institute of Technology Gumi 39177 Republic of Korea; ^2^ Department of Chemical Engineering Pohang University of Science and Technology Pohang 37673 Republic of Korea; ^3^ Department of Polymer Science and Engineering Department of Energy Engineering Convergence Kumoh National Institute of Technology Gumi 39177 Republic of Korea; ^4^ Department of Applied Bioengineering Graduate School of Convergence Science and Technology Seoul National University Seoul 08826 Republic of Korea; ^5^ Department of Chemical and Biological Engineering and Institute of Chemical Processes College of Engineering Seoul National University Seoul 08826 Republic of Korea; ^6^ Advanced Institute of Convergence Technology Suwon 16229 Republic of Korea; ^7^ Department of Nanoenergy Engineering Pusan National University Busan 46241 Republic of Korea; ^8^ Department of Chemical and Biomolecular Engineering Seoul National University of Science and Technology Seoul 01811 Republic of Korea

**Keywords:** artificial synapse, electrolyte‐gated transistor, long‐term plasticity, neuromorphic computing, side chain

## Abstract

Interest has grown in services that consume a significant amount of energy, such as large language models (LLMs), and research is being conducted worldwide on synaptic devices for neuromorphic hardware. However, various complex processes are problematic for the implementation of synaptic properties. Here, synaptic characteristics are implemented through a novel method, namely side chain control of conjugated polymers. The developed devices exhibit the characteristics of the biological brain, especially spike‐timing‐dependent plasticity (STDP), high‐pass filtering, and long‐term potentiation/depression (LTP/D). Moreover, the fabricated synaptic devices show enhanced nonvolatile characteristics, such as long retention time (≈10^2^ s), high ratio of *G*_max_/*G*_min_, high linearity, and reliable cyclic endurance (≈10^3^ pulses). This study presents a new pathway for next‐generation neuromorphic computing by modulating conjugated polymers with side chain control, thereby achieving high‐performance synaptic properties.

## Graphical abstract

In organic semiconductors‐based neuromorphic devices, it is difficult to endow long‐term plasticity in diketopyrrolopyrrole (DPP) polymers due to insufficient interaction with ions. In this article, a rational way is proposed to overcome the deficiency of nonvolatile properties by tailoring the length of the alkyl side‐chain of DPP polymers.

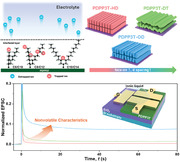

## 1 Introduction

Large‐scale artificial intelligence models and cloud services have highlighted the importance of energy‐efficient computing hardware to replace traditional devices based on the von Neumann architecture.^[^ ^1^ ^]^ In particular, von Neumann structures with separate central processing units and memory create bottlenecks^[^ ^2^, ^3^ ^]^ that limit computational speed and significantly increase energy usage when complex or unstructured data are processed.^[^ ^4^ ^]^ In contrast, neuromorphic computing, inspired by the human brain structure, where memory and logic reside in a single unit^[^ ^5^ ^]^ offers a fundamentally different architecture that enables parallel computation, has low power consumption, and efficient information processing.^[^ ^6^, ^7^ ^]^ Therefore, such neuromorphic architectures mimicking the characteristics of the brain have received considerable attention, and a need for synaptic devices that fulfill the role of synapses has been recognized.

Several types of devices such as memristors and memtransistors have been proposed to implement synaptic behaviors.^[^ ^8^, ^9^, ^10^, ^11^ ^]^ Memristors possess memory and switching functions, making them suitable for use in artificial synapses mimicking neurotransmission.^[^ ^12^, ^13^, ^14^, ^15^ ^]^ However, owing to structural limitations and sneak‐path problems,^[^ ^16^, ^17^ ^]^ neural transmission cannot be controlled precisely by external stimuli. Instead, memtransistors, which are three‐terminal devices with extra controllability, enabling efficient biofunctionalization^[^ ^18^ ^]^ with a wide range of electrical modulation,^[^ ^19^ ^]^ making them very promising for artificial synapses.^[^ ^20^, ^21^ ^]^ In particular, electrolyte‐gated transistors (EGTs), which implement the behavior of neurotransmitters through ions,^[^ ^22^ ^]^ enable dynamic synapses,^[^ ^23^ ^]^ the information‐processing functions of neuronal spikes,^[^ ^24^ ^]^ and synaptic weight control through electrochemical modulation.^[^ ^25^ ^]^ Combined with these advantages, organic‐based EGTs have been widely investigated^[^ ^26^, ^27^, ^28^ ^]^ due to their low cost, scalability, and tunable physical and chemical properties. However, most of the reported research with polymers has utilized the heterostructure of the polymer or additional treatment such as solvent or heat treatment to achieve synaptic characteristics, which have been considered a potential problem.^[^ ^29^, ^30^, ^31^ ^]^

To overcome these challenges, the structural deformation or physicochemical property modulation of single polymers has been used to control the electrical properties of devices by modifying the band structure and redistributing the electron density without additional processing.^[^ ^32^, ^33^, ^34^ ^]^ Among them, DPP‐based conjugated polymers have been extensively studied due to their advantages such as low cost, flexibility, and tunable photoelectric properties.^[^ ^35^, ^36^ ^]^ Li et al. observed that the orientation of diketopyrrolopyrrole (DPP) polymers changes the electrical properties including transfer behavior and carrier mobility.^[^ ^37^ ^]^ To control this orientation, DPP‐based polymers typically modulate the packing texture through functional substituents such as side chains or conjugated segments.^[^ ^38^, ^39^, ^40^, ^41^ ^]^ Despite these attempts, DPP is not easily electrochemically doped by anion dopants, resulting in short‐term plasticity (STP) or short retention time, which limits its use in artificial synapse implementation.^[^ ^42^, ^43^ ^]^ To achieve non‐volatile characteristics such as long‐term plasticity (LTP) for advanced neuromorphic computing, it is required to be able to modulate the interaction between the channel layer and the ions.

Here, we present a rational novel strategy to simultaneously control the adsorption energy for ions and orientation of polymers through the modulation of side chains in polymers. We fabricated EGTs with PDPP3T‐based polymers as the channel layer and diethylmethyl(2‐methoxyethyl)ammonium bis(trifluoromethylsulfonyl)imide (DEME‐TFSI) as the electrolyte. The fabricated device exhibited an enhanced nonvolatile retention time (≈10^2^ s) and successfully mimicked the biological behaviors of the human brain, such as spike‐timing dependent‐plasticity (STDP), high‐pass filtering, and long‐term potentiation/depression (LTP/D). In addition, the device showed a high ratio of *G*_max_/*G*_min_, high linearity, and reliable stability in maintaining the LTP/D characteristics for ≈10^3^ pulses. Furthermore, our synaptic transistor showed high accuracy (≈94.03%) in Modified National Institute of Standards and Technology database (MNIST) recognition simulations. These results not only indicate that the biological synapse‐like properties were realized completely but also shed light on next‐generation neuromorphic computing.

## 2 Results and Discussion

**Figure 1:**
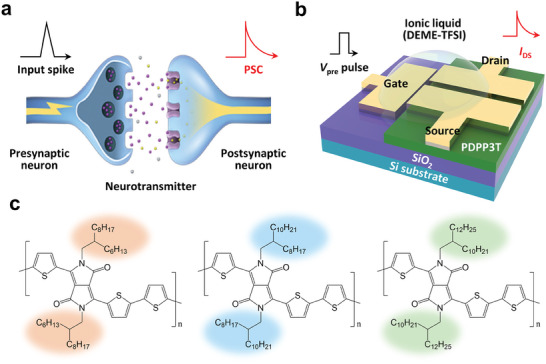
Schematic illustration of a) synapse in a biological brain, and b) the structure of fabricated 3‐terminal synaptic device using PDPP3T‐based polymers on the SiO_2_/Si substrate. c) Polymer structures of PDPP3T‐HD having C6/C10‐branched alkyl length (left), PDPP3T‐OD having C8/C12‐branched alkyl length (middle), and PDPP3T‐DT having C10/C14‐brancced alkyl length (right).

**Figure** **1**a shows the unit structure of the biological synapses in the human brain. A unit synapse comprises three main components: a presynaptic neuron, postsynaptic neuron, and neurotransmitter that electrochemically connects two adjacent neurons. First, a presynaptic neuron generates a potential difference in the form of a spike that triggers the release of a neurotransmitter from the presynaptic neuron. Subsequently, a postsynaptic current (PSC) is generated after the receptor in the postsynaptic neuron receives the neurotransmitter, which allows continuous transmission of the electrical signal. The generated PSC depends on the number of neurotransmitters released by the presynaptic neuron and the history of the presynaptic spikes. These basic principles allow synapses to perform various biological functions, such as STP, LTP, and STDP by changing the synaptic weight.

The EGTs were fabricated on SiO_2_/Si substrates to demonstrate their biological functions (Figure 1b). The fabricated devices consisted of a gate electrode to apply a presynaptic pulse, source, and drain (S/D) electrodes, which were deposited using a thermal evaporator, PDPP3T‐based polymers acting as postsynaptic neurons, and DEME‐TFSI as the neurotransmitter. The channel width (*W*) and length (*L*) of the fabricated device are 800 and 100 µm, respectively (Figure S1, Supporting Information). To investigate the effect of alkyl chain lengths on the synaptic characteristics, we prepared three distinct polymers: PDPP3T‐HD, PDPP3T‐OD, and PDPP3T‐DT, featuring alkyl chains of 2‐hexyldecyl, 2‐octyldodecyl, and 2‐decyltetradecyl, respectively. In each case, the alkyl length was increased by two carbon atoms while maintaining the backbone (Figure 1c).

To observe the electrical properties of our fabricated devices, we generated transfer curves (Figure S3, Supporting Information). A clear clockwise hysteresis is a typical characteristic due to the mobile ions in ionic liquids, and it was verified as the largest in PDPP3T‐OD. From these results, we could approximate the threshold voltage of PDPP3T polymers‐based devices at above –2 V. To investigate how side‐chain control affects synaptic behavior, we examined the change in channel current in response to a pulsed voltage. First, we measured the channel current in response to a single pulse, which represents STP (Figure S4, Supporting Information). It was applied presynaptic voltage (*V*_pre_) from –1 to –4 V with a pulse width of 20 ms to the synaptic devices. The highest excitatory postsynaptic current (EPSC) peaked at *V*_pre_ of –4 V in all three fabricated synaptic devices and, in the case of PDPP3T‐OD, the channel current was maintained slightly, with a gradual decrease.

**Figure 2:**
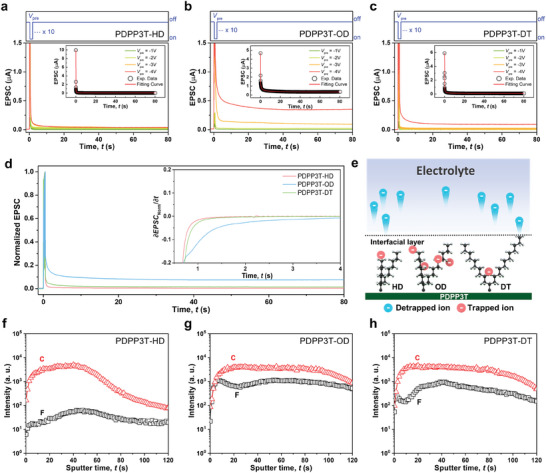
LTP characteristics of PDPP3T‐based synaptic transistors. EPSC responses to 10 sequential pulses (20 ms width and 20 ms interval) with different amplitudes in a) PDPP3T‐HD, b) PDPP3T‐OD, and c) PDPP3T‐DT. Inset: SEF fitting curve from the immediate end of the pulses when *V*_pre_ = −4 V. d) Normalized EPSC curve of a PDPP3T‐based synaptic transistor applied with a gate voltage of −4 V. Inset: First derivatives of each EPSC curve. e) Schematic illustration of detrapped/trapped ions at the interface between PDPP3T polymers and electrolytes. TOF‐SIMS depth profiles (intensity as function of sputter time) of carbon and fluorine in f) PDPP3T‐HD, g) PDPP3T‐OD, and h) PDPP3T‐DT films after applying gate bias of −4 V amplitude.

Subsequently, to analyze the LTP characteristics, we applied 10 consecutive pulses of different amplitudes (*V*_pre_ = −1, −2, −3, and −4 V) at a fixed pulse width (20 ms), interval (20 ms), and *V*_DS_ = −1 V to the synaptic devices (**Figure** **2**a–c). When 10 consecutive pulses were applied to both the PDPP3T‐HD and the PDPP3T‐DT synaptic devices, the EPSC value increased rapidly and then tended to revert to the initial current value after *V*_pre_ was turned off. Interestingly, the PDPP3T‐OD synaptic device clearly retained a nonvolatile channel current of ≈10% of the EPSC peak current at *V*_pre_ = −4 V even after the transient time. This behavior was also observed to some extent at *V*_pre_ = −3 V but, at lower voltages, the nonvolatile current slowly decreased and returned to its initial value, i.e. it did not exhibit LTP property anymore. To analyze the retention behavior of EPSCs by the continuous electrical pulses was analyzed by fitting the following Kohlrausch stretched exponential function (SEF):

(1) $$I\;\left(t\right)=I_{0}\;\exp\left[-{\left(\frac{t}{\tau }\right)}^{\beta }\right]+I_{\infty }$$

where *I*(t) is the relaxation function, *I*_0_ is the pre‐exponential factor, *I*_∞_ is the EPSC level after the decay, τ is the relaxation time, and β is stretching index ranging from 0 to 1.^[^ ^44^, ^45^ ^]^ The τ value of PDPP3T‐OD is 25 ms, which is longer than ‐HD (1 ms) or ‐DT (5 ms). This increase in relaxation time indicates a transition from STP to LTP with an increase in retention time, which represents a more stable enhancement of synaptic weight and a lower rate of forgetting.

For further comparison for the observed LTP characteristics, the EPSC value at *V*_pre_ = −4 V of each synaptic device was normalized (Figure 2d). The PDPP3T‐OD synaptic device maintained a nonvolatile retention current ratio (≈10 times larger) for a longer time than the PDPP3T‐HD and PDPP3T‐DT synaptic devices. To observe the kinetic variation from each normalized EPSC curve, we verified the trend through the first derivative and found that the transient time was longer for the PDPP3T‐OD synaptic device than for the PDPP3T‐HD and PDPP3T‐DT synaptic devices. These results could be attributed to the working mechanisms of the EGTs. In general, EGTs exhibit electrical modulation through the migration of ions by the electric field of the gate electrode, with the anions accumulating at the interface between the electrolyte and the channels of the devices. When the electric field disappeared, the channel current gradually disappeared through the diffusion of ions back into the electrolyte. This finding indicated that PDPP3T‐OD accumulated a larger number of TFSI anions than PDPP3T‐HD and PDPP3T‐DT. Moreover, from the obtained LTP characteristics of the PDPP3T‐OD synaptic device, it was assumed that most ions were bound despite the back diffusion of a large number of TFSI anions and the disappearance of the electric field. To illustrate this finding, in the case of PDPP3T‐OD, most de‐trapped ions diffused into the electrolyte; however, some anions were retained (Figure 2e). When electrical pulses are applied to the gate, the TFSI anions migrate to the polymer/electrolyte interface and, subsequently, the ions penetrate the polymer region, and the current increases exponentially. After the pulse sequence, they diffuse back out and the current returns to its original value. However, the TFSI anions were adsorbed onto the alkyl chain and had not yet returned; therefore, the current was maintained. These electrical properties were not strongly influenced by the crystallinity of the polymer, as shown by the melting enthalpies of PDPP3T‐OD and DT not differing significantly (Figure S4, Supporting Information).

Furthermore, to validate our approach, we conducted a chemical analysis of the electrolyte/PDPP3T‐based polymer interface using time‐of‐flight secondary‐ion mass spectroscopy (TOF‐SIMS) after applying pulses of −4 V (Figure 2f–h). In the TOF‐SIMS depth profile, because of the effect of the applied pulse, PDPP3T‐OD exhibited the highest fluorine intensity among the PDPP3T‐based polymers across the sputtering time. The observed intensity for fluorine was attributed to TFSI anions, which were adsorbed from the electrolyte onto the polymer owing to the electrical influence. We demonstrated that the measured current change was attributable to the ions, showing that the highest number of TFSI anions remained trapped even after the gate voltage was turned off. This was similar to the PDPP3T‐OD‐based device showing the highest percentage of retention behavior in the LTP measurements. The strong intensity observed for carbon indicated that the measurement for the polymer was correct.

**Figure 3:**
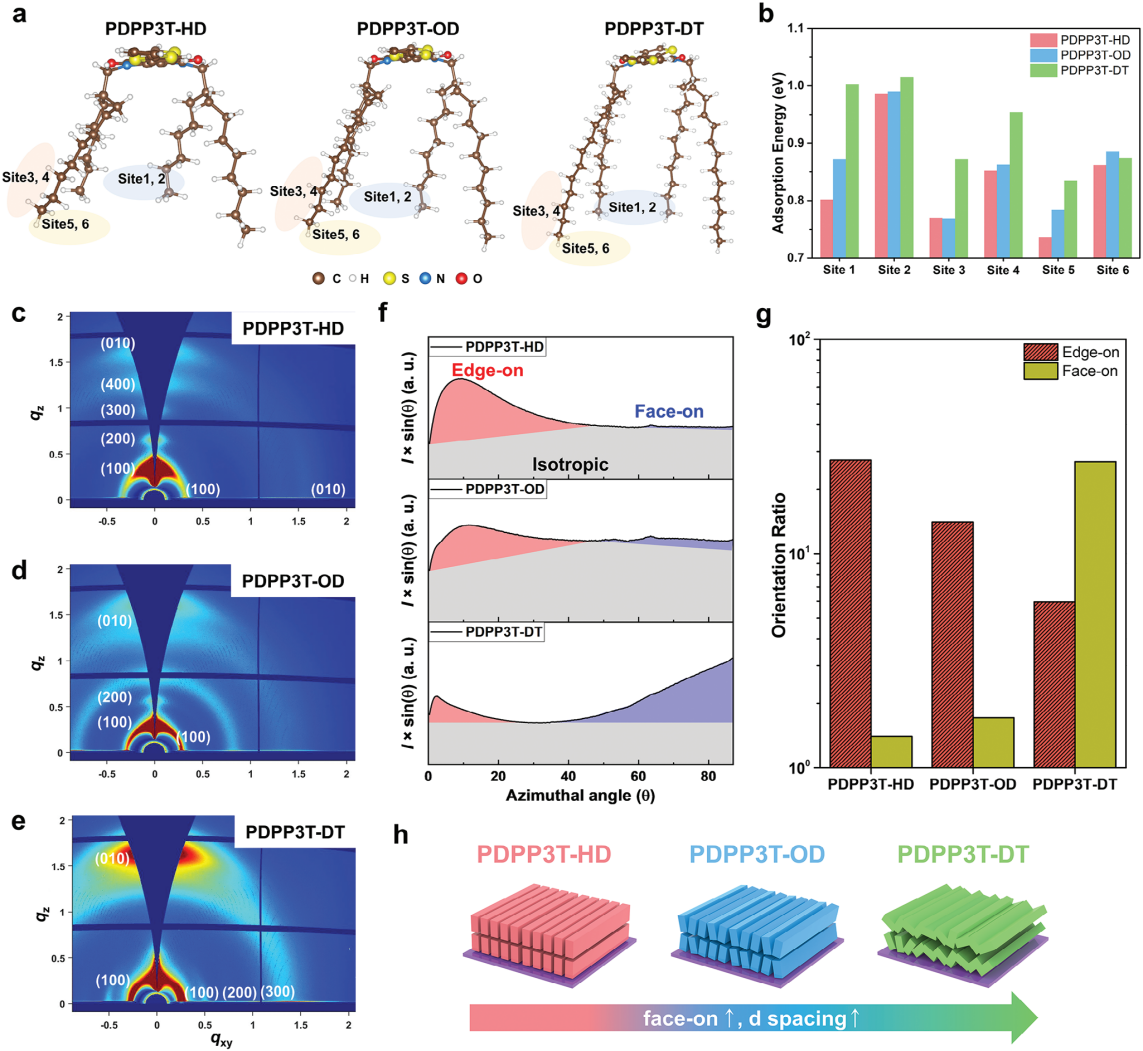
a) Accessible adsorption sites for anion in the side chains of PDPP3T‐HD (left), PDPP3T‐OD (middle), and PDPP3T‐DT (right). b) Comparisons of adsorption energy of anions at different adsorption sites. Obtained 2D‐GIWAXS patterns of c) PDPP3T‐HD, d) PDPP3T‐OD, and e) PDPP3T‐DT. f) Azimuthal integrations of the PDPP3T‐HD (top), PDPP3T‐OD (middle), and PDPP3T‐DT (bottom) from GIWAXS data. g) Orientation ratio comparisons between edge‐on and face‐on polymer directions. h) Schematic illustration of the favorable orientations of each polymer. Preference for face‐on directionality and d‐spacing increases.

Density functional theory (DFT) calculations were performed to further analyze the adsorption behavior of TFSI. To investigate the influence of the side chains in PDPP3T‐HD, ‐OD, and ‐DT, we considered the monomer as a model system. The PDPP3T‐HD, ‐OD, and ‐DT model systems consist of C_50_N_2_O_2_S_3_H_74_, C_58_N_2_O_2_S_3_H_90_, and C_66_N_2_O_2_S_3_H_106_ atoms, respectively. For systematic calculation of the adsorption behavior of the TFSI anion, we considered three locations on the side chain, namely the outer, lower, and inner regions as potential adsorption sites. Furthermore, even for the same site, we considered whether the oxygen or fluorine part of the anion was adsorbed, resulting in six different calculated adsorption energies (**Figure** **3**a). The binding energies exhibited by PDPP3T‐HD, PDPP3T‐OD, and PDPP3T‐DT were stronger at sites 2, 4, and 6 than at sites 1, 3, and 5 (Figure 3b). Observation of the molecular structure of TFSI clearly showed that in comparison with fluorine, oxygen exhibited a stronger adsorption tendency toward the PDPP3T chain (Figures S5–S7, Supporting Information). Furthermore, the interaction with oxygen increased in strength as the length of the chain increased, as evidenced by the exchange of more electrons, leading to enhanced adsorption capacity (Figure S8, Supporting Information). As the chain length increases, the presence of hydrogen in the structure strengthens the adsorption capacity of anions. The highest adsorption energy was observed at site 2, which corresponds to the inner region among the adsorption sites, providing evidence for robust adsorption of multiple hydrogen atoms in the chain and oxygen atoms in TFSI. The DFT results revealed the adsorption mechanism within the local structure, and when the monomer became an actual polymer, the microscale structure altered the adsorption characteristics.

Grazing incidence wide‐angle X‐ray scattering (GIWAXS) was performed to investigate molecular packing and crystal orientation within the semi‐crystalline PDPP3T‐based thin films. Figure 3c–e shows the 2D‐GIWAXS patterns of PDPP3T‐HD, ‐OD, and ‐DT, respectively. Table S2 (Supporting Information) describes the information along the out‐of‐plane (OoP, *θ* = 0) and in‐plane (IP, *θ* = 90) parameters, including the peak position with *d*‐spacing and full width at half maximum (FWHM), with the crystalline coherence length (CCL) obtained from the 2D‐GIWAXS patterns. The (010) diffraction peak in patterns indicated 𝜋–𝜋 stacking of conjugated backbones. This corresponds to face‐on orientation when the (010) diffraction peak showed in OoP direction with stacked lamella (h00) structures in the IP direction. Conversely, the edge‐on orientation could be determined with 𝜋–𝜋 stacking along the IP direction with stacked lamella (h00) in the OoP direction. As shown in Figure S9 (Supporting Information), the distances of observed peaks were measured carefully at *q_xy_* = 0.30, 0.28, and 0.25 Å^−1^ for PDPP3T‐HD, PDPP3T‐OD, and PDPP3T‐DT films, with corresponding lamella d‐spacing values of 1.94, 2.25, and 2.44 nm, respectively. These results implied that the increased side‐chain length widened the distance between lamellas. The CCL was derived from the Scherrer equation to analyze the crystalline domain size in films. The resulting CCLs for PDPP3T‐HD, PDPP3T‐OD, and PDPP3T‐DT were 4.77, 11.42, and 12.48 nm along the IP direction, respectively, and the observed trend of increased domain size facilitated intermolecular charge carrier transport properties. In PDPP3T‐based thin films, all crystalline regions were preferentially mixed with face‐on and edge‐on orientation. We systematically calculated the population ratio between the edge‐on and face‐on crystallites (Figure 3f). For the shortest side chain, PDPP3T‐HD showed a dominant edge‐on orientation compared with face‐on orientation. As the side chains gradually lengthened, the preferable crystallite orientation changed to face‐on, i.e., side chains are not exposed on the surface (Figure 3g). To explain our experimental results, we present a schematic drawing that shows the orientation of crystallites depends on the length of the side chain (Figure 3h).

**Figure 4:**
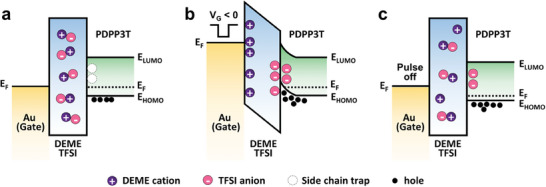
Energy band diagram of gate/ionic liquid/PDPP3T‐OD in the synaptic device a) at equilibrium, b) programming, and c) pulse‐off states.

To understand the mechanism underlying synaptic characteristics, an energy‐band diagram was constructed (**Figure** **4**). The results showed that the binding of TFSI anions to the side chains of PDPP3T‐OD and the nonvolatile characteristics could be explained by the energy band diagram. First, at equilibrium, the alignment of the fermi levels of the Au electrode and PDPP3T‐OD occurred when DEME‐TFSI functioned as a gate dielectric layer within the device structure (Figure 4a). As no electric field was applied to the PDPP3T‐OD semiconductor, no TFSI anions were bound. When a negative voltage above −3 V was applied to the device in equilibrium, the TFSI anion in the electric double layer bordering the PDPP3T‐OD started to be strongly embedded in the side‐chain trap, as shown in Figure 4b. The embedded TFSI anion freed the holes inside PDPP3T‐OD to contribute to the conduction mechanism which, in turn, allowed the TFSI anion to maintain a constant excited current even in the pulse‐off state because it does not diffuse back to its original state (Figure 4c). This interpretation based on the energy band diagram is not only consistent with Figure 2g but also supports the finding that TFSI anions do not diffuse back into the bulk electrolyte from the interior of the PDPP3T‐OD film, even in the pulse‐off state.

To further confirm the nature of our synaptic device we also demonstrated the characteristics of PPF, which is a typical short‐term plastic behavior. This behavior occurs when a synapse is successively activated by two sequences of stimuli, resulting in an amplified response to the second stimulus because of facilitation by the Ca^2+^ residue around the channel by the first stimulus.^[^ ^46^ ^]^ The PPF index is defined as the ratio of the peak EPSC (A_1_) caused by the first pulse applied to the gate to the peak EPSC (A_2_) caused by the subsequent second pulse.

(2) $$\mathrm{PPF}\;\mathrm{index}=A_{2}/A_{1}$$

**Figure 5:**
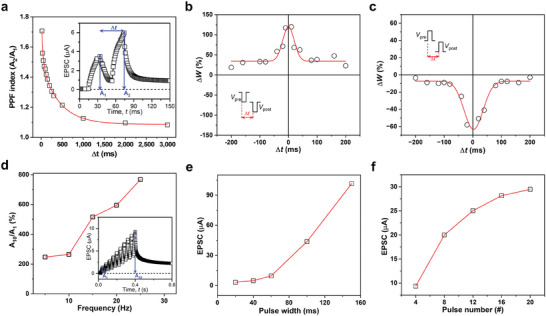
Robustness of PDPP3T‐OD synaptic transistors. a) Plot of PPF index, defined as the ratio of A_2_/A_1_, as a function of pulse interval time (∆*t*) between two consecutive pulses (−4 V, 20 ms). Inset: Conductance changes with adjacent applied pulses, with an interval of 20 ms. Experimental demonstration of spike‐timing‐dependent plasticity (STDP) using PDPP3T‐OD‐based device. b) STDP demonstrating Hebbian and c) anti‐Hebbian learning. (Inset: Illustrate the pre‐and postsynaptic voltage pulses in the STDP measurements.) d) Current gain, defined as ratio of A_10_/A_1_, plotted as a function of frequency from 5 to 25 Hz showing high‐pass filtering characteristics of synaptic device. Inset: ESPC change by 10 pulses with a frequency of 25 Hz. e) Changed EPSC at the 10th pulse by applying 10 consecutive pulses with different pulse widths, and f) Changed EPSC after applying a different number of pulses to synaptic transistors.

The PPF was demonstrated by applying two consecutive electrical pulses with different interval times (Δ*t*) of a square waveform of 20 ms and −4 V to the gate electrodes at the synaptic devices (Figure S10a, Supporting Information). As the pulse interval time increased from 20 to 3000 ms, the PPF index decreased from 1.71 to 1.08, indicating that enhancement of the EPSC peak in PDPP3T‐OD decreased as the interval between pulses increased (**Figure** **5**a). The relationship between the PPF index and interval time can be fitted with a double‐exponential function.

(3) $$\mathrm{PPF}=C_{0}+C_{1}\exp\left(-\frac{\Delta t}{\tau_{1}}\right)+C_{2}\exp\left(-\frac{\Delta t}{\tau_{2}}\right)$$

where C_0_ is a constant, C_1_ and C_2_ are the initial facilitation values, with C_1_ is the value in the slow phase and C_2_ is the value in the rapid phase, Δ*t* is the pulse interval time, and τ_1_, τ_2_ are the PPF relaxation times in the slow and rapid phases, respectively. Through fitting to Equation 2, we obtained C_0_ = 1.09, τ_1_ = 35.62 ms, and τ_2_ = 431.67 ms for the PDPP3T‐OD device. In the biological synapse, when C_0_ = 1, the relaxation times of each phase (τ_1_ and τ_2_) are tens and hundreds of ms.^[^ ^47^ ^]^ The results of these fittings confirmed that the PPF behavior of PDPP3T‐OD devices was significantly more similar to biological synapses than that of other PDPP3T‐based devices (Figures S12a and S13a, Supporting Information). To further explore the possibility of low energy consumption of our device, we observed the PPF behavior under lower drain voltage (Figure S11, Supporting Information). The energy consumption was calculated using the equation *E*  =  *AIW*.^[^ ^48^, ^49^ ^]^ *A*  =   − 0.1 mV is the drain voltage, *I*  =  593 nA is the current flowing across the device, and *W*  =  20 ms is the width of the electrical pulse. The *E* obtained from the PDPP3T‐OD‐based artificial synapses was ≈1.18 pJ per spike, which is similar to previous studies and shows a low level of energy consumption.^[^ ^43^, ^50^, ^51^ ^]^ This means that our devices also have the potential to mimic the low energy consumption of biological synapses.

In biological synapses, the temporal factor of the stimulus can determine the synaptic weight. STDP is a key synaptic function in biological synaptic mimicking and represents changes in the weight of the synapse as a function of the relative time between the pre‐ and post‐synaptic spikes.^[^ ^52^, ^53^ ^]^ To implement STDP behavior, we applied pulses by varying the time interval (∆*t*) between spikes. We denote ∆*t* > 0 when the presynaptic spike is applied before the postsynaptic spike and ∆*t* < 0 when the postsynaptic spike is applied first. The current measured over the ∆*t* was calculated as the synaptic weight change using the following equation. ∆*W* = *W*_STDP−_ *W*_0_/*W*_0_ × 100,^[^ ^54^, ^55^ ^]^ where ∆*W* is the synaptic weight change and *W*_0_ and *W*_STDP_ are the current values due to the pre‐ and postsynaptic spikes, respectively. Figure 5b,c shows the change in ∆*W* with respect to ∆*t*. When we applied only potentiation pulses (−4 V), we saw Hebbian learning that decreased symmetrically with respect to ∆*t*, with positive ∆*W* at all times. Applying depression pulses (+2 V) only showed anti‐Hebbian learning with a negative ∆W and a symmetric decrease on the y‐axis. All the above results indicate that the STDP behavior can be simulated by our fabricated PDPP3T‐OD devices.

Synapses can act as filters that amplify the transmission rate based on the signal frequency. High‐pass filtering occurs in characteristic synapses, in which the synapse responds strongly to signals with frequencies above a certain level and weakly or not at all to signals with frequencies below that level.^[^ ^56^, ^57^ ^]^ We mimicked high‐pass filtering by calculating the EPSC gain when 10 series of pulses (−4 V, 20 ms) with frequencies between 5 and 25 Hz were applied to the gate electrode (Figure S10b, Supporting Information). The EPSC gain was calculated as the ratio of the peak EPSC for the 10th pulse (A_10_) to the peak EPSC for the 1st pulse (A_1_). At 25 Hz, the EPSC gain increased to 290% (Figure 5d).

To further investigate the other synaptic characteristics of our device, we observed the change in the EPSC when pulses were applied to the gate electrode with different pulse widths and pulse numbers (Figure S10c,d, Supporting Information). When 10 consecutive pulses with an amplitude of −4 V were applied while modulating the pulse width, the EPSC change increased from 3.35 to 101.386 µA as the width increased from 20 to 150 ms (Figure 5e). In addition, we observed that the EPSC change became three times larger as the number of pulses applied to the gate electrode increased (Figure 5f). These results demonstrated that modulation of the pulse width and pulse number could enhance the memory level by controlling the movement of TFSI anions to the interface of the electrolyte/PDPP3T‐based polymer and channel.

**Figure 6:**
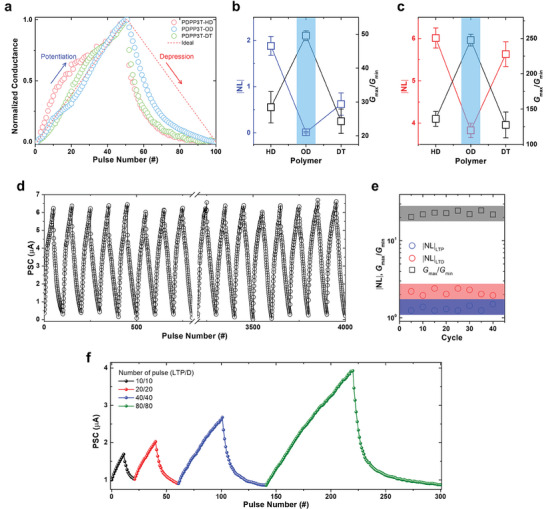
a) Normalized LTP and LTD characteristics of PDPP3T polymer synaptic transistors under application of 50/50 potentiation (−4 V, 20 ms)/depression (+2 V, 20 ms) pulses. Plots of *G*_max_/*G*_min_, and ∣*NL*∣ values depending on branched alkyl length in b) LTP region and c) LTD region. d) Cyclic endurance of LTP and LTD curves, and e) plots of *G*_max_/*G*_min_, and ∣*NL*∣ values for 4000 pulse numbers. f) LTP and LTD measurements of PDPP3T‐OD synaptic transistors under application of various pulse sets (10, 20, 40, and 80 pulses).

In synaptic devices, factors such as the dynamic range (*G*_max_/*G*_min_) and nonlinearity (*NL*) of LTP/D characteristics are considered crucial properties for neuromorphic computing. These factors significantly influence the determination of the learning configuration and accuracy as a parameter in the neural network.^[^ ^58^, ^59^ ^]^ Therefore, we observed the LTP/D properties of devices with different alkyl chain lengths of PDPP3T‐based polymers and investigated the changes in properties with chain length (Figure S14a–c, Supporting Information). This was measured by 50 potentiation pulses (−4 V, 20 ms) and 50 subsequent depression pulses (+2 V, 20 ms) under *V*_DS_ = −1.0 V. A comparison of the normalized LTP/D profiles depending on the side chain indicated that the PDPP3T‐OD‐based synaptic devices most closely resembled the ideal curve (**Figure** **6**a). The NL was calculated from the normalized LTP/D curve using the following equations^[^ ^58^ ^]^

(4) $$G_{\mathrm{LTP}}=B\left(1-e^{\left(-\frac{P}{A}\right)}\right)+G_{\min}$$

(5) $$G_{\mathrm{LTD}}=-B\left(1-e^{\left(\frac{P-P_{\max}}{A}\right)}\right)+G_{\max}$$

(6) $$B=\left(G_{\max}-G_{\min}\right)/\left(1-e^{\frac{-P_{\max}}{A}}\right)$$

where *G*_LTP_ and *G*_LTD_ denote the conductance of potentiation and depression, respectively, *P*_max_ is the maximum number of pulses, and *A* is a variable that controls the nonlinear behavior of potentiation and depression by adjusting the *G*_LTP_/*G*_LTD_ curve to determine the *NL* value by fitting the measured LTP/D curve (Figure S14d–f, Supporting Information). We plotted the *NL* and *G*_max_/*G*_min_ values as functions of the alkyl chain length of the PDPP3T‐based polymers in the LTP and LTD regions (Figure 6b,c). In both regions, the PDPP3T‐OD‐based synaptic devices with C8/C10 branched alkyl chains showed the lowest *NL* values and the largest *G*_max_/*G*_min_ values. These results suggested that medium‐length PDPP3T‐OD polymers are optimal for synaptic devices, as nonvolatile memories with high linearity and large dynamic range show improved accuracy in artificial neural network (ANN).^[^ ^58^, ^60^ ^]^

Accordingly, we applied 4000 pulses (40 cycles) to confirm the stability and reliability of the PDPP3T‐OD synaptic device (Figure 6d). Our device showed reliable LTP/D behavior without significant changes during 4000 pulses (Figure S15, Supporting Information). Moreover, the major factors *NL* values, *G*_max_/*G*_min_ values remained stable over 40 cycles (Figure 6e). We found that the *NL* values for the LTP and LTD regions remained constant below 2 and 3, respectively, and that the dynamic range remained in a range of tens. Furthermore, we increased the number of pulses applied to the device to 20, 40, 80, and 160 and observed that the PSC increased when the number of pulses in a cycle increased (Figure 6e).

**Figure 7:**
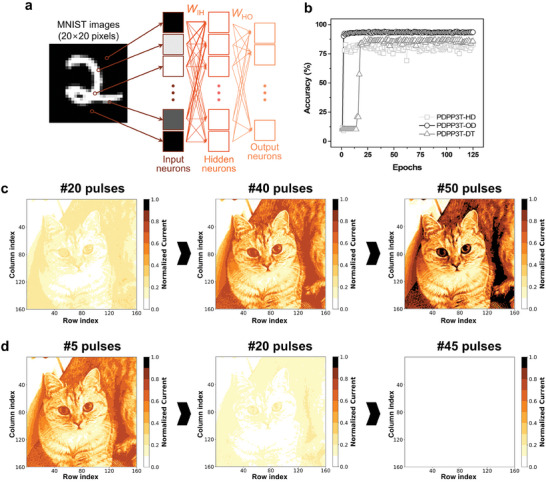
Simulation of PDPP3T‐OD synaptic devices for neuromorphic computing. a) Scheme of MLP neural network comprising 400 input neurons, 100 hidden neurons, and 10 output neurons. b) The recognition accuracy for each epoch depends on PDPP3T polymers. A 160 × 160 synapse array is utilized to store and erase images. A real image of a cat is encoded by appointing electrical pulses to each 160 × 160 pixels. The detected image data after c) the 20th, 40th, and 50th pulse processed with PDPP3T‐OD under potentiated pulses (−4 V) and d) the 5th, 20th, and 45th pulse processed with PDPP3T‐OD under depressed pulses (+2 V).

Finally, supervised learning was performed on MNIST handwritten digit images to measure the accomplishment of synaptic devices based on our PDPP3T polymer. To perform the simulation through a multilayer perceptron (MLP)‐based ANN, the *NL* value, *G*_max_/*G*_min_, and conductance for each pulse were employed. The PDPP3T polymer synaptic device‐based ANN is a two‐layer perceptron that consists of 400 input neurons, 100 hidden neurons, and 10 output neurons (**Figure** **7**a). The 400 input neurons represented the 20 × 20 MNIST data, while the 10 output neurons represented groups of numbers from zero to nine. When the accuracy of handwritten digit recognition was measured according to the side‐chain length of the PDPP3T polymer, PDPP3T‐OD exhibited a higher recognition rate of 94.03% at the 110th epoch than PDPP3T‐HD and PDPP3T‐DT, which showed accuracies of less than 90% (Figure 7b). The results indicated that the recognition performance could be enhanced substantially by controlling the behavior of TFSI anions through modulation of the length of the alkyl chain of the polymer. Subsequently, image storage and forgetting processes were performed using coded cat pictures of 160 × 160 pixels based on the PDPP3T‐OD device. The images were recognized as potentiation pulses and depression pulses were applied to the PDPP3T‐OD synaptic device. We confirmed that applying 20, 40, and 50 potentiation pulses led to gradually stronger recognition of the image, and applying 5, 20, and 45 depression pulses led to forgetting the image (Figure 7c,d). Therefore, the results suggested that our alkyl chain‐modified synaptic devices have potential applications in image recognition, storage, and forgetting through the modulation of electrical signals in neuromorphic systems.

## 3 Conclusion

In conclusion, we investigated synaptic properties such as LTP and LTP/D through side chain modulation of conjugated polymers. The electrical properties of the fabricated devices were successfully controlled by the side chain of the polymers used as channel layers without further processing. The properties of our synaptic devices are similar to those of biological synapses such as PPF, high‐pass filtering, and LTP. They also exhibited excellent retention properties, a high *G*_max_ */G*_min_ ratio, good linearity, and reliable durability. Moreover, the recognition accuracy of handwritten digits in the MNIST database was 94.03% by using multilayer ANN. This study proposes the possibility of implementing artificial synapses for next‐generation neuromorphic computing by realizing synaptic performance through side chain engineering in polymer‐based synaptic devices.

## 4 Experimental Section

### Materials

Thiophene was purchased from TCI; Trimethyltin chloride from Sigma‐Aldrich; 3,6‐bis(5‐bromothiophenyl)−2,5‐bis(2‐octyldodecyl) pyrrolopyrrole‐1,4‐dione from SunaTech Inc; 3,6‐bis(5‐bromothiophenyl)−2,5‐bis(2‐decyltetradecyl) pyrrolopyrrole‐1,4‐dione from Angene Chemical (Ann Arbor, MI, USA) and all monomers were used without further purification; PDPP3T‐HD from Ossila (Sheffield, UK) (*M*_n_ = 30.4 kg mol^−1^ and *Ð* = 2.77); and the ionic liquid diethylmethyl (2‐methoxyethyl) ammonium bis(trifluoromethylsulfonyl)imide (DEME‐TFSI) from Sigma‐Aldrich.

### Synthesis

A 2, 5‐bis(trimethylstannyl)thiophene was synthesized employing a lithiation process based on a previously reported procedure.^[^ ^52^, ^53^, ^54^ ^]^ In brief, thiophene (1 eq, 0.3697 g, 4.39 mmol) and TMEDA (2.1 eq, 1.0710 g, 9.22 mmol) were dissolved in 20 mL anhydrous hexane at 0 °C. Subsequently, n‐butyllithium (5.8 mL, 9.22 mmol, 1.6 m in hexane) was admixed with the solution. The mixture underwent reflux at 70 °C for 45 min and then was cooled to 0 °C. Trimethyltin chloride (9.3 mL, 9.22 mmol, 1 m in hexane) was slowly dropped into the mixture. The mixture was stirred overnight at room temperature. The resulting solution was quenched with deionized water, extracted with hexane, and washed several times with water. The organic layer was dried over magnesium sulfate, and the volatiles were removed in vacuum chamber. Finally, the crude product was recrystallized using methanol.^[^ ^52^ ^]^ Yield: 1.108 g (62%). PDPP3T‐OD was synthesized through stille polycondensation. The 2, 5 bis(trimethylstannyl)thiophene (1 eq, 0.120 g, 0.293 mmol) and 3, 6‐bis(5‐bromothiophenyl)−2, 5‐bis(2‐octyldodecyl)pyrrolopyrrole‐1, 4‐diene (1 eq, 0.297 g, 0.293 mmol) were taken to dissolve in 10 mL degassed toluene. Tri(*o*‐tolyl) phosphine (0.08 eq) and Pd_2_(dba)_3_ (0.02 eq) were added and allowed to stir overnight. To purify the polymer, the resulting solution was separated in methanol, hexane, and chloroform. The chloroform fraction was captured for device construction. Yield: 254 mg (94%), *M*_n_ = 62.6 kg mol^−1^, and *Ð* = 3.28. PDPP3T‐DT was synthesized employing the stille polymerization process described above using 3, 6‐bis(5‐bromothiophenyl)−2, 5‐bis(2‐decyltetradecyl) pyrrolpyrrole‐1, 4‐dione (1 eq, 0.332 g, 0.293 mmol) as a monomer instead of 3, 6‐bis(5‐bromothiophenyl)−2, 5‐bis(2‐octyldodecyl) pyrrolopyrrole‐1, 4‐dione. The monomers were used at the same scale. Yield: 239 mg (87%), *M*_n_ = 38.8 kg mol^−1^, and *Ð* = 3.34.

### Device Fabrications

The synaptic device was fabricated on a boron‐doped p‐type Si wafer covered with a thermally grown 285 nm thick SiO_2_ dielectric layer. The wafers used for device fabrication were cut to a size of 1.5 cm × 1.5 cm. The wafer was cleaned by sonication in isopropyl alcohol (IPA) and acetone for 20 min, and subsequently washed with ethanol and deionized water. After blow drying with nitrogen gas, the wafers were exposed to UVO at 250 °C for 5 min to modify the surface properties. The solutions for spin coating were prepared by dissolving the PDPP3T‐based polymers at 7 mg mL^−1^ in chlorobenzene and stirred at 120 °C for 30 min. The PDPP3T solutions were spin‐coated onto SiO_2_ substrates as a channel layer for 60 s at 1500 rpm. The coated polymer channel layers were dry etched twice by reactive ion etching (RIE) using a Femto Science (Republic of Korea) covance vacuum plasma system with pure argon gas at 200 W for 3 min, and covered with a metal shadow mask. The first etching removed the polymer below the gate region, and the second left the polymer material only in the channel region between the source and drain. For the gate, source, and drain electrodes, 40 nm thick gold was deposited on the PDPP3T channel layer at 10^−6^ torr using a thermal evaporator. DEME‐TFSI was dropped 0.2 µL through a micropipette between the gate, source, and drain electrodes.

### Computational Details

The Vienna Ab‐initio Simulation Package (VASP)^[^ ^61^, ^62^, ^63^ ^]^ was employed for conducting ab initio DFT calculations to investigate the adsorption behavior. The exchange‐correlation effects were modeled using the Perdew‐Burke‐Ernzerhof (PBE) functional,^[^ ^64^ ^]^ which was derived from the Generalized Gradient Approximation (GGA). The energy cutoff for the plane wave was 400 eV, and the Projector Augmented Wave (PAW).^[^ ^65^ ^]^ The energy convergence threshold was set to 10^–4^ eV, and the force convergence criterion was maintained at 0.05 eV Å^−1^ during the calculations. The Gamma 1 × 1 × 1 K‐point was selected for geometric optimization of the monomer structures. The PDPP3T‐HD, OD, and DT structures were modeled within a 30 Å × 30 Å × 30 Å cell.

### Measurement and Analysis

The electrical properties of the synaptic devices were measured using a Keithley 4200A‐SCS (Keithley Instruments, OH, USA) semiconductor analyzer in the dark condition at room temperature with 10^−3^ A current compliance. GIWAXS measurements were performed at the Pohang Accelerator Laboratory (PAL) Pohang, Korea, employing synchrotron radiation at beamline 3C.

### Simulation of Neural Networks

ANN simulation was performed on a Linux system using the open‐source program NeuroSim ver. 3.0. To calculate recognition accuracy, a multilayer perceptron consisting of 400 input neurons, 125 hidden neurons, and 10 output neurons was used. The input neurons of the ANN were 20 × 20 MNIST digits binarized black‐and‐white patterns for handwritten images. The dynamic range, read voltage, number of conductance levels, *NL* value, device‐to‐device/cycle‐to‐cycle variation, write pulse voltage, and width were used as parameters to measure the real device in the simulation. For 60 000 training images, training with 20 000 training images per epoch for a total of 125 epochs was performed and, subsequently, measured recognition accuracy on 10000 test images.

## Conflict of Interest

The authors declare no conflict of interest.

## Author Contributions

J.S. and S.C. contributed equally to this work. J.S., Y.J., W.L., and E.L. newly introduced PDPP3T‐based synaptic devices. J.S., H.J., D.L., D.J., and E.L. designed and fabricated devices. J.S., J.K., C.L., and Y.S.K. performed neuromorphic computing and simulation of PDPP3T‐based synaptic devices. Y.J., S.C., K.C., J.K., and W.L. discussed the selection of materials and the theoretical developments. J.S., Y.J., S.C., J.K., and E.L. wrote the manuscript based on discussion with all authors. J.K., W.L., and E.L. supervised the project direction including experimental and theoretical investigations in this study.

## Supporting information

Supporting Information

## Data Availability

The data that support the findings of this study are available from the corresponding author upon reasonable request.
