# Supplementary material for: Unveiling the Role of Side Chain for Improving Nonvolatile Characteristics of Conjugated Polymers‐Based Artificial Synapse
Source: Adv Sci (Weinh). 2024 Feb 26;11(16):2400304. doi: 10.1002/advs.202400304 (PMC11040376; doi:10.1002/advs.202400304)
Supplement: Supplementary file 1 — Supporting Information [file ADVS-11-2400304-s001.pdf]

ADVANCED SCIENCE
Open Access

Supporting Information

for *Adv. Sci.*, DOI 10.1002/advs.202400304

Unveiling the Role of Side Chain for Improving Nonvolatile Characteristics of Conjugated Polymers-Based Artificial Synapse

Junho Sung, Sein Chung, Yongchan Jang, Hyoik Jang, Jiyeon Kim, Chan Lee, Donghwa Lee, Dongyeong Jeong, Kilwon Cho, Youn Sang Kim, Joonhee Kang, Wonho Lee* and Eunho Lee**

Supporting Information

Unveiling the Role of Side Chain for Improving Nonvolatile Characteristics of Conjugated Polymers-Based Artificial Synapse

Junho Sung[a†], Sein Chung[b†], Yongchan Jang[c], Hyoik Jang[a], Jiyeon Kim[d], Chan Lee[e], Donghwa Lee[a], Dongyeong Jeong[a], Kilwon Cho[b], Youn Sang Kim[d,e,f], Joonhee Kang[g], Wonho Lee[c]*, and Eunho Lee[h]**

[a]Department of Chemical Engineering, Kumoh National Institute of Technology, Gumi 39177, Republic of Korea

[b]Department of Chemical Engineering, Pohang University of Science and Technology, Pohang 37673, Republic of Korea

[c]Department of Polymer Science and Engineering, Department of Energy Engineering Convergence, Kumoh National Institute of Technology, Gumi 39177, Republic of Korea

[d]Department of Applied Bioengineering, Graduate School of Convergence Science and Technology, Seoul National University, 1 Gwanak-ro, Gwanak-gu, Seoul 08826, South Korea

[e]Department of Chemical and Biological Engineering, and Institute of Chemical Processes, College of Engineering, Seoul National University, Gwanak-ro 1, Gwanak-gu, Seoul 08826, Republic of Korea

[f]Advanced Institute of Convergence Technology, 145 Gwanggyo-ro, Yeongtong-gu, Suwon, 16229, Republic of Korea

[g]Department of Nanoenergy Engineering, Pusan National University, Busan 46241, Republic of Korea

[h]Department of Chemical and Biomolecular Engineering, Seoul National University of Science and Technology, Seoul 01811, Republic of Korea

[†]These authors equally contributed to this work.

***E-mail**: j.kang@pusan.ac.kr, 1holee@kumoh.ac.kr, ehl@seoultech.ac.kr

Keywords: neuromorphic computing, electrolyte-gated transistor, long-term plasticity, artificial synapse, side chain

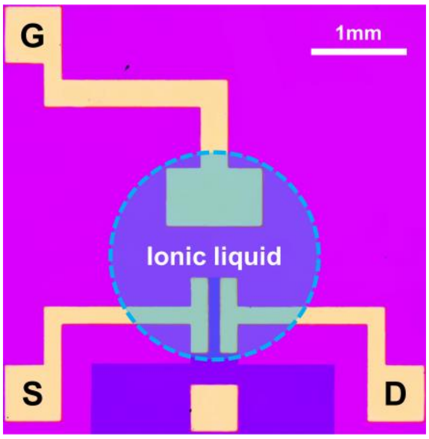

Figure S1. An optical image of the fabricated PDPP3T-based synaptic transistors on the SiO_2/Si substrate. The blue circle indicates the droplet region of the ionic liquid.

The polymer film was spin-coated using PDPP3T solution on SiO_2/Si substrate as a wafer scale. The film was patterned by RIE and the gate, source, and drain electrodes were deposited by thermal evaporation. The above image shows the device fabricated on a SiO_2/Si substrate measuring 1.5 cm × 1.5 cm.

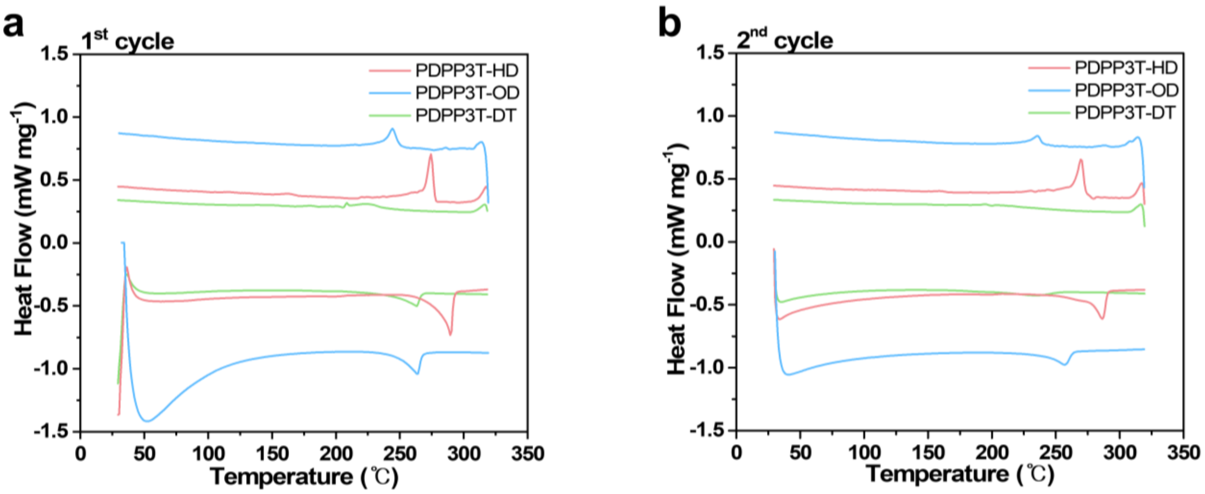

Figure S2. Differential scanning calorimetry of PDPP3T polymers for a) 1st cycle b) 2nd cycle.

Polymer	1st cycle		2nd cycle	
	Melting point (°C)	Melting enthalpy (J g^{-1})	Melting point (°C)	Melting enthalpy (J g^{-1})
PDPP3T-HD	289	21.94	286	12.14
PDPP3T-OD	264	11.83	257	7.96
PDPP3T-DT	263	9.15	233	3.82

Table S1. Melting point and melting enthalpy for cycle according to polymer.

Differential scanning calorimetry (DSC) of PDPP3T series shows an endotherm with peak at the melting temperature (230–290 °C) with enthalpy of fusion.[1] From PDPP3T-HD to OD and DT, the melting point tends to decrease in both 1- and 2-cycles due to the effect of the longer alkyl side chain.[2] The decrease in melting point and enthalpy of PDPP3T-DT (with a long chain) is more pronounced because of the large effect of structural relaxation.[3] As a result, the melting enthalpy of PDPP3T-DT is the lowest at 3.82 J g^{-1}, which showing that the degree of crystallization is low due to the long side chain.

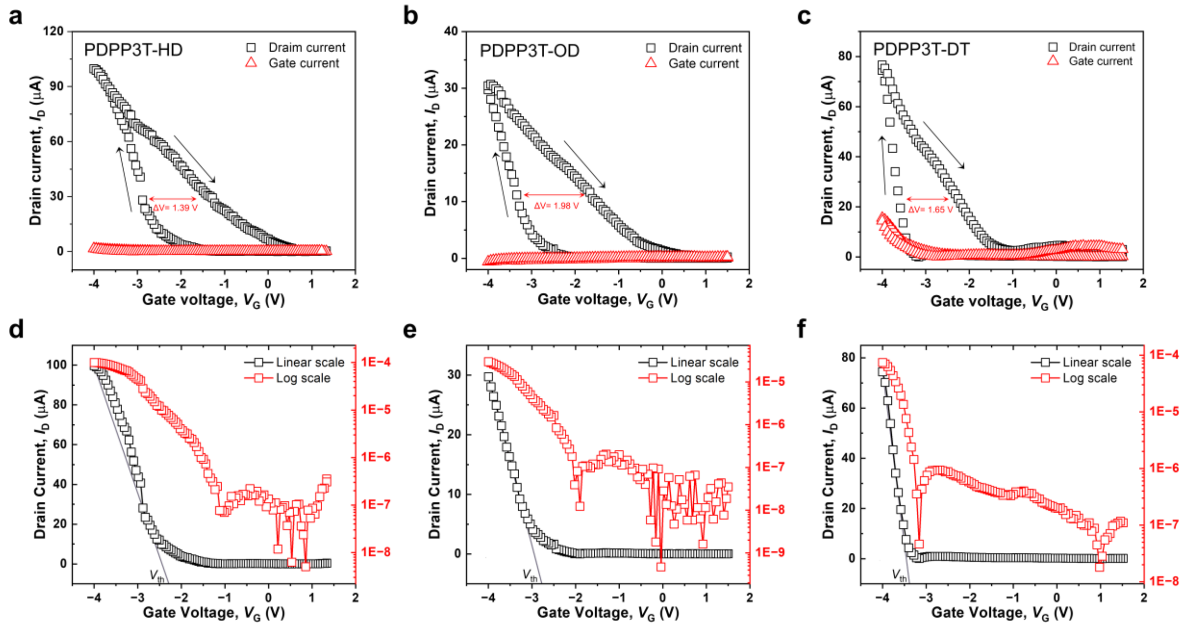

Figure S3. Hysteresis analysis. Transfer curves of a) PDPP3T-HD, b) PDPP3T-OD, c) PDPP3T-DT in linear scale. Clockwise hysteresis characteristics are clearly observed in three polymers. Forward sweep of d) -HD, e) -OD, and f) -DT with linear scale (black) and $\log_{10}$ scale (red).

Sample	Slope of I_D/V_G	*W* (μm)	*L* (μm)	V_D (V)	cap/*A* (F/cm^2)	Mobility, μ (cm^{2}V^{-1}s^{-1})	*SS* (mV/dec)
PDPP3T-HD	-5.87E-05	800	200	-1	1.00E-06	1.47E+01	0.105779
PDPP3T-OD	-2.65E-05	800	200	-1	1.00E-06	6.63E+00	0.090148
PDPP3T-DT	-4.37E-05	800	200	-1	1.00E-06	1.09E+01	0.0989

Table S2. The values used to calculate mobility and subthreshold swing (*SS*), as well as the calculated values.

We fabricated a synaptic device depending on PDPP3T-HD, PDPP3T-OD, and PDPP3T-DT and obtained the transfer curve. From this we could see that the hysteresis becomes wider depending on the branched alkyl chain length. With the forward sweep we could get threshold voltage and factors for calculating mobility, and subthreshold swing. Using these values, we calculated that HD has the largest mobility and OD has the smallest *SS*.

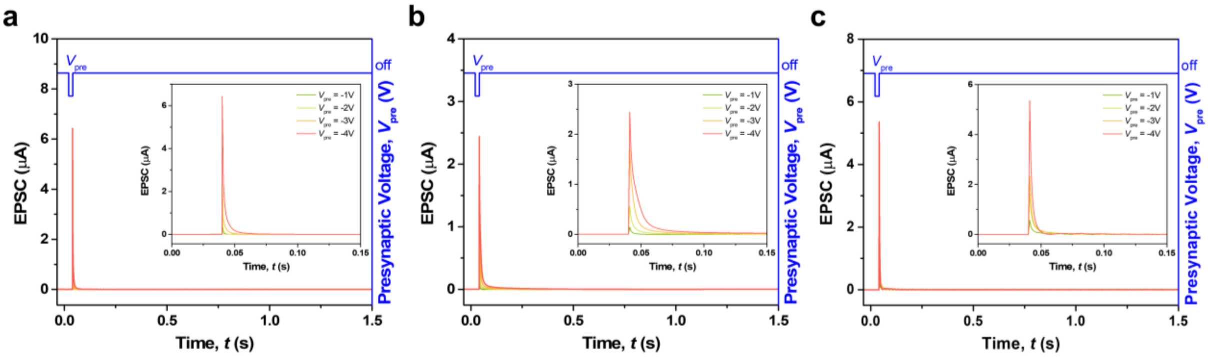

Figure S4. STP characteristics of PDPP3T-based synaptic transistor. EPSC responses to single pulse (20 ms width) with different amplitudes in a) PDPP3T-HD, b) PDPP3T-OD, and c) PDPP3T-DT. Input: Magnified view of the base current region.

As the drain voltage is -1 V and a 20 ms pulse is applied according to the gate voltage, the STP characteristics can be adjusted according to the PDPP3T material. Depending on the side chain length, the current reduction speed of OD is the slowest, followed by DT and HD. It can be seen that the EPSC with increasing as the gate voltage increases.

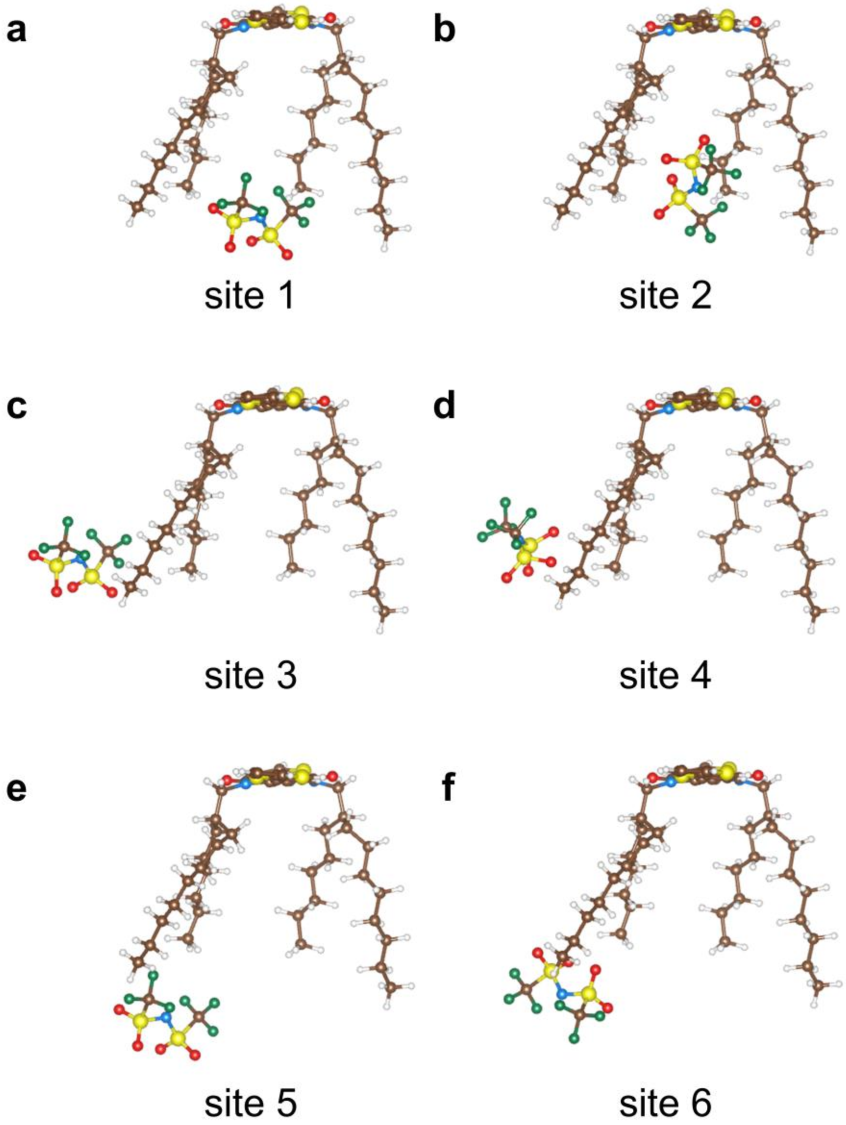

Figure S5. Schematic illustrations for the adsorption energy calculation of TFSI anion at site 1 to site 6 (a–f) in PDPP3T-HD.

The figure above indicates where the TFSI anion is able to adsorb on the PDPP3T-HD material. It can be seen that it can adsorb on both sites 1–6.

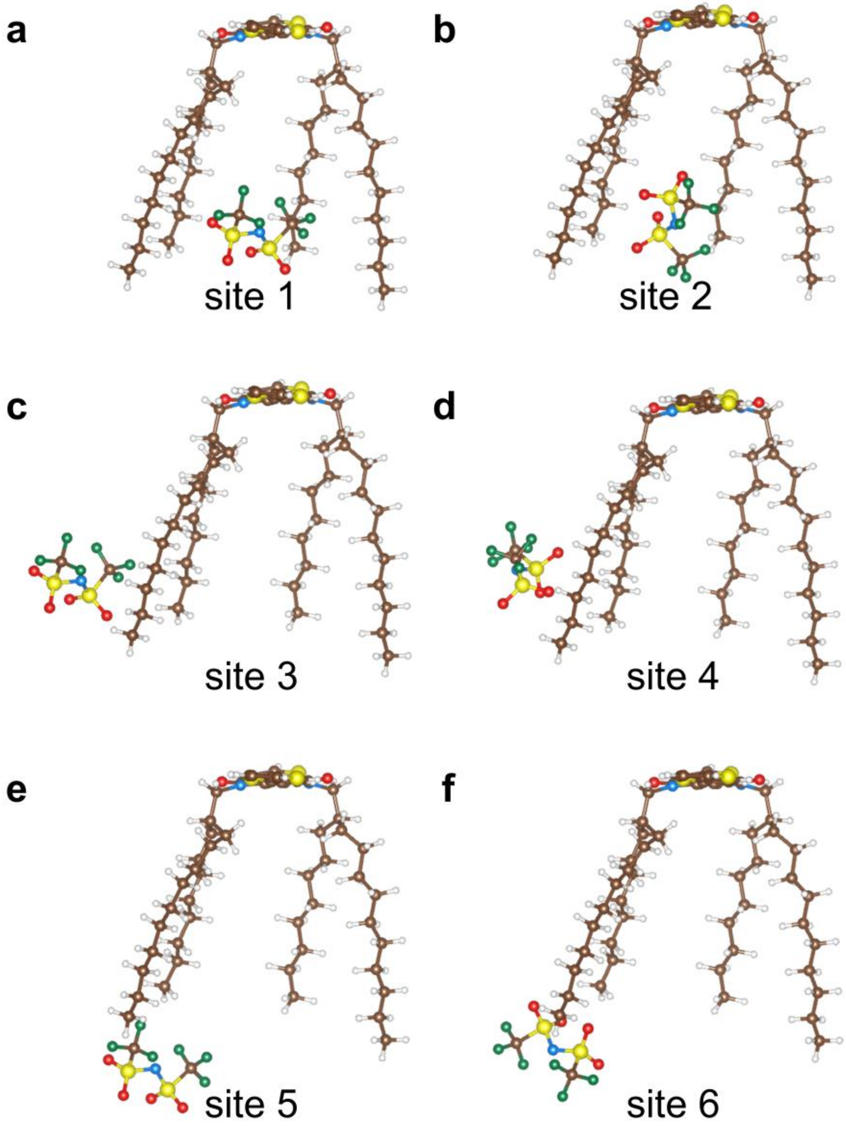

Figure S6. Schematic illustrations of the calculation of the adsorption energy of the TFSI anion at site 1 to site 6 (a–f) in PDPP3T-OD.

The above image shows where the TFSI anion can adsorb on the PDPP3T-OD material. It can be seen that there can be adsorption on both sites 1–6.

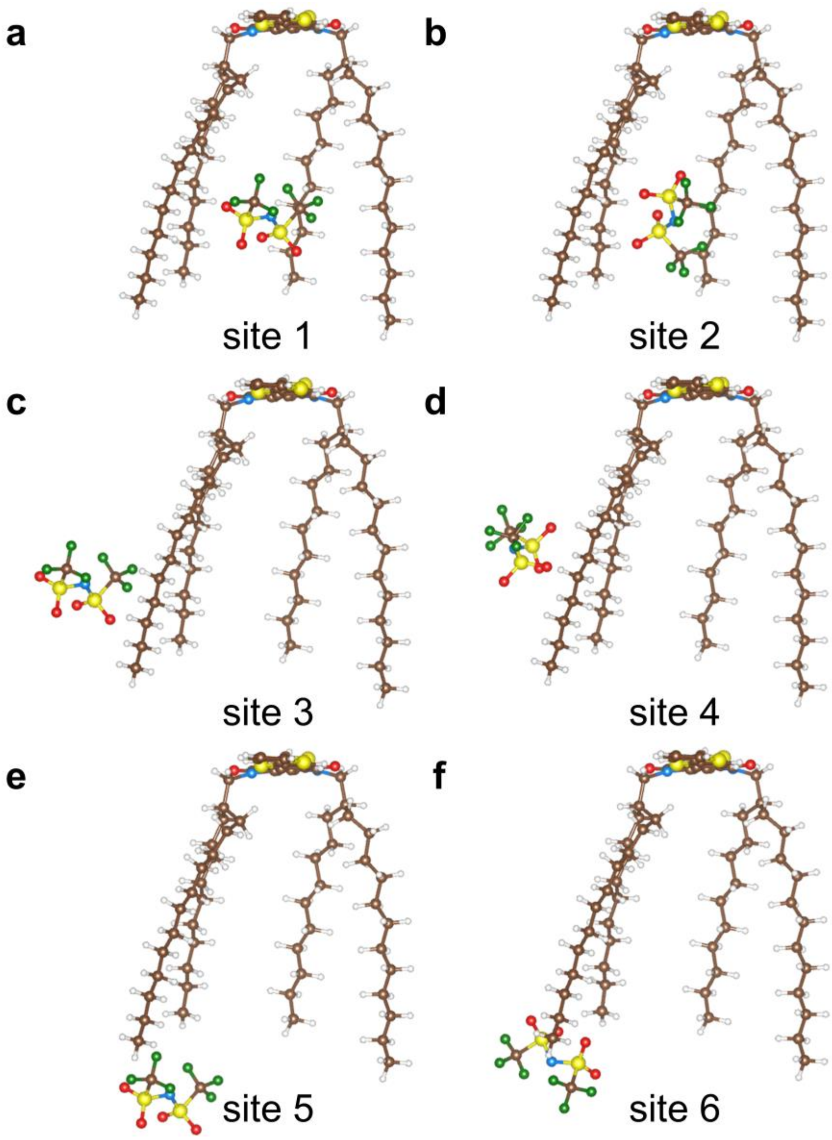

Figure S7. Schematic of adsorption energy calculation of the TFSI anion at site 1 to site 6 (a–f) in PDPP3T-DT.

The above image shows where the TFSI anion can adsorb on the PDPP3T-DT material. It can be seen that it is able to adsorb on both of the sites 1–6.

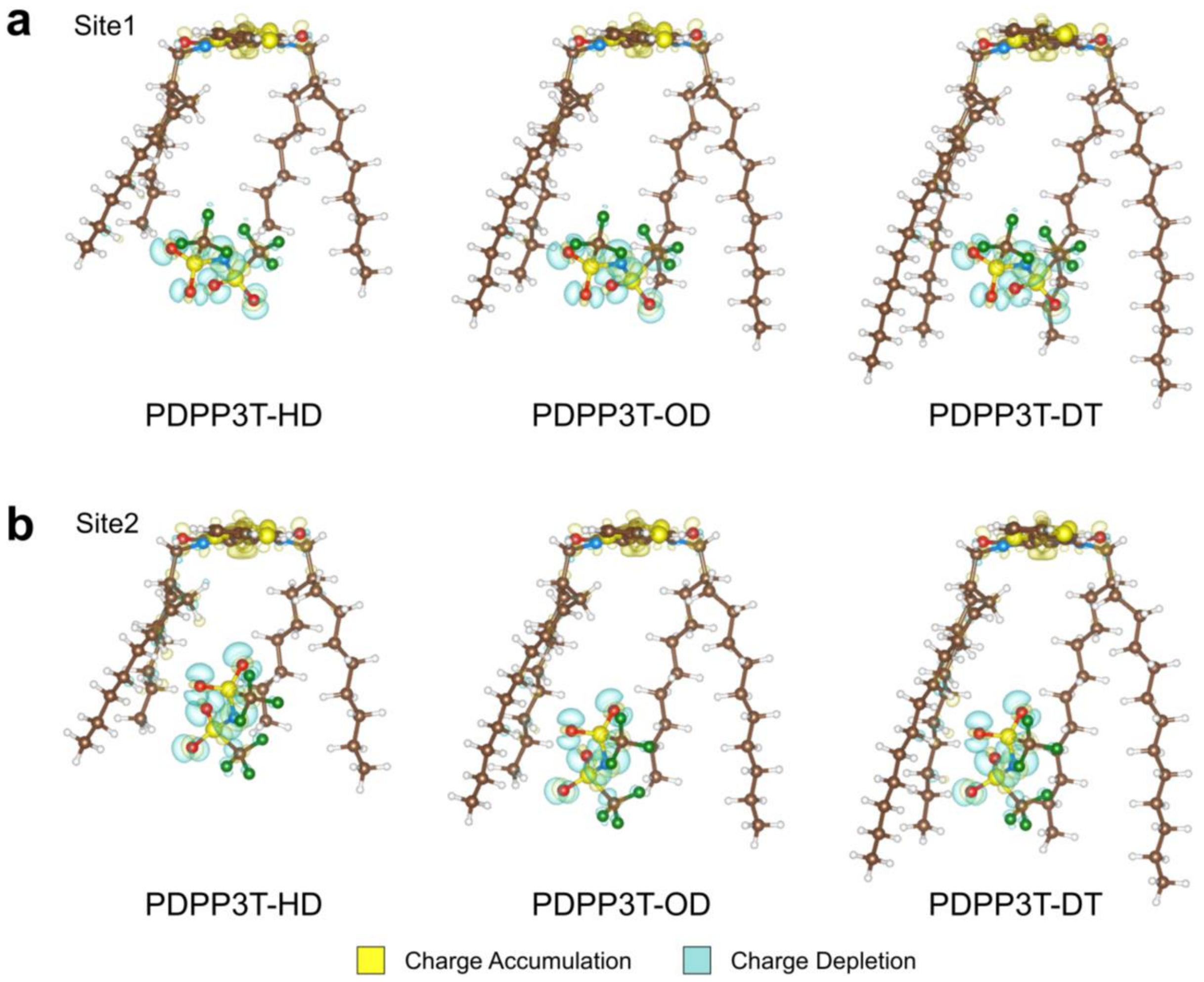

Figure S8. The charge density difference between PDPP3T and TFSI anion for site 1 and 2. The unit of charge density is 0.002 e/Å^3.

The image above illustrates which part of the TFSI anion is involved in strong interactions, exchanging electrons with PDPP3T. We can identify that the oxygen within TFSI primarily engages in exchanging electrons with the PDPP3T chain.

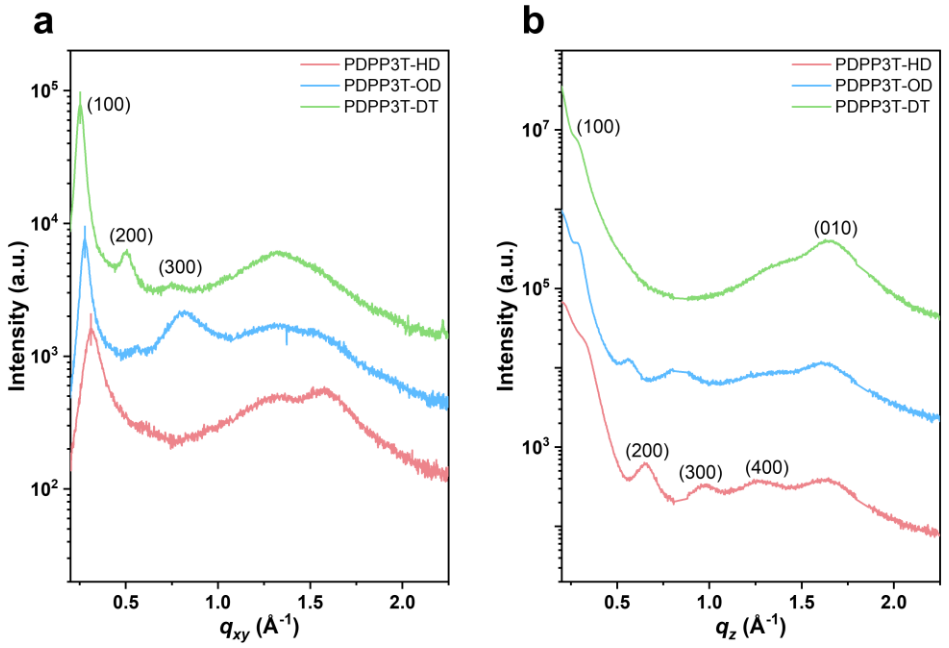

Figure S9. 1D-GIWAXS data of a) in-plane and b) out-of-plane for PDPP3T-HD (red), PDPP3T-OD (blue), and PDPP3T-DT (green).

	In-plane (100)	
	d-spacing (nm)	CCL (Å) K = 1
PDPP3T-HD	1.94	47.68
PDPP3T-OD	2.25	114.23
PDPP3T-DT	2.44	124.81

Table S3. d-spacing and CCL of PDPP3T based polymers.

The π-π stacking (010) and lamella (h00) reflection (*d*-spacing) obtained by the equation of $d = 2\pi/q$, where q is the corresponding x-coordinate of maximum diffraction peak intensity. The CCL was calculated quantitatively by using the Scherrer equation: CCL = $2\pi K/\Delta q$, where K is the form factor (K = 1.0), and Δq is the FWHM of each diffraction peak. The crystallites orientation was calculated through integration of (100) and (010) diffraction peak with calibration by multiplying intensity with $sin(\theta)$ according to azimuthal angle. (out-of-plane (OoP, $\theta = 0^{\circ}$) and in-plane (IP, $\theta = 90^{\circ}$))

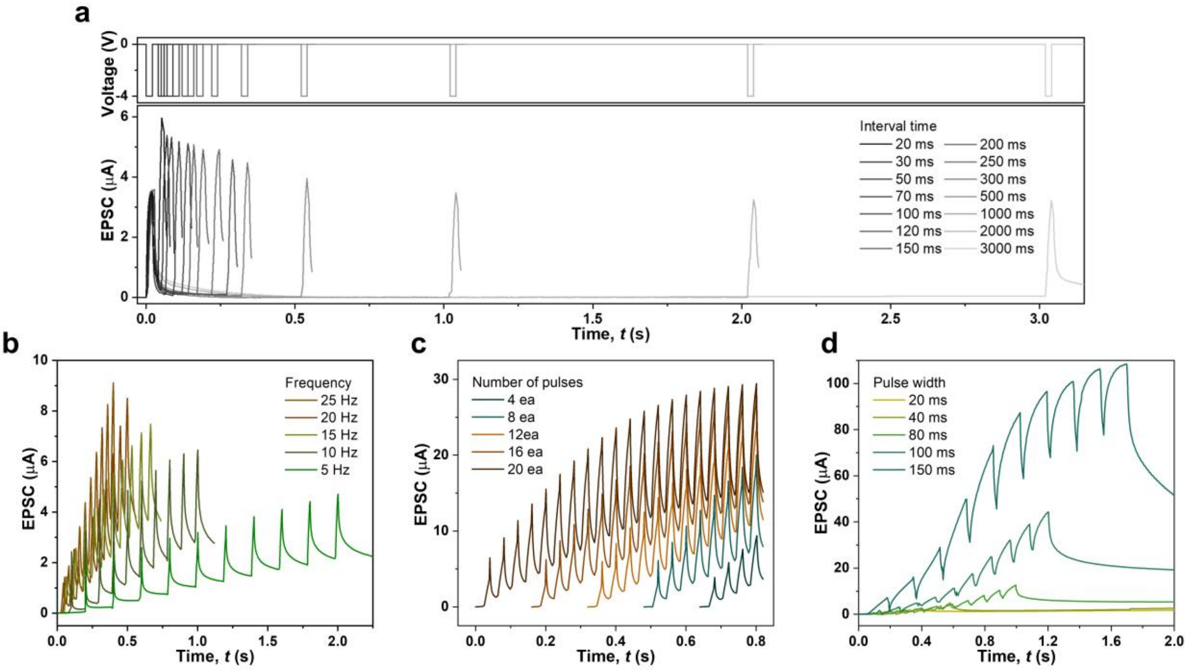

Figure S10. Synaptic characteristics of PDPP3T-OD synaptic devices. a) Change in EPSC over actual time as pulse interval increases. The change in EPSC as a function of time by b) pulse input frequency, c) pulse number, and d) pulse width when applying a series of pulses.

Figure S10 exhibits additional synaptic properties of PDPP3T-OD synaptic devices. Figure S10a shows that increasing the interval between two consecutive pulses decreases the EPSC to a second changing pulse. Figure S10b-d indicate that the EPSC response varies with changes in frequency, number of pulses, and pulse width, respectively.

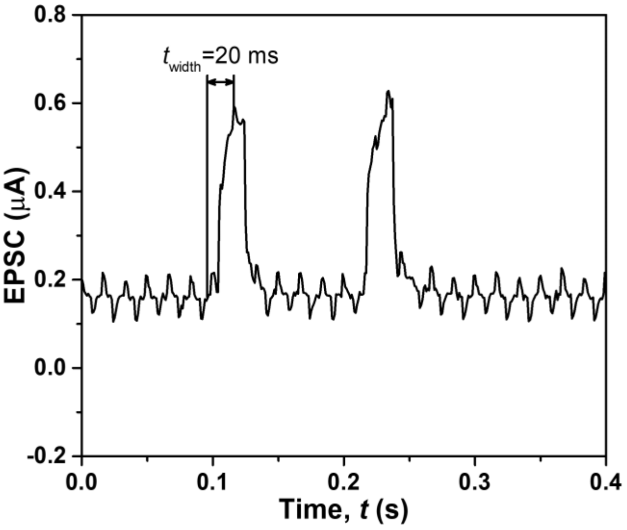

Figure S11. EPSC triggered by an even lower drain voltage (V_{DS} = -0.1 mV) and short-term synaptic enhancement by two consecutive electrical pulses (-4 V, 20 ms).

Figure S11 shows PPF behavior under low drain voltage to achieve low energy-consumption with PDPP3T-OD devices. As the results we observed that *E* was ~1.18 pJ per spike.

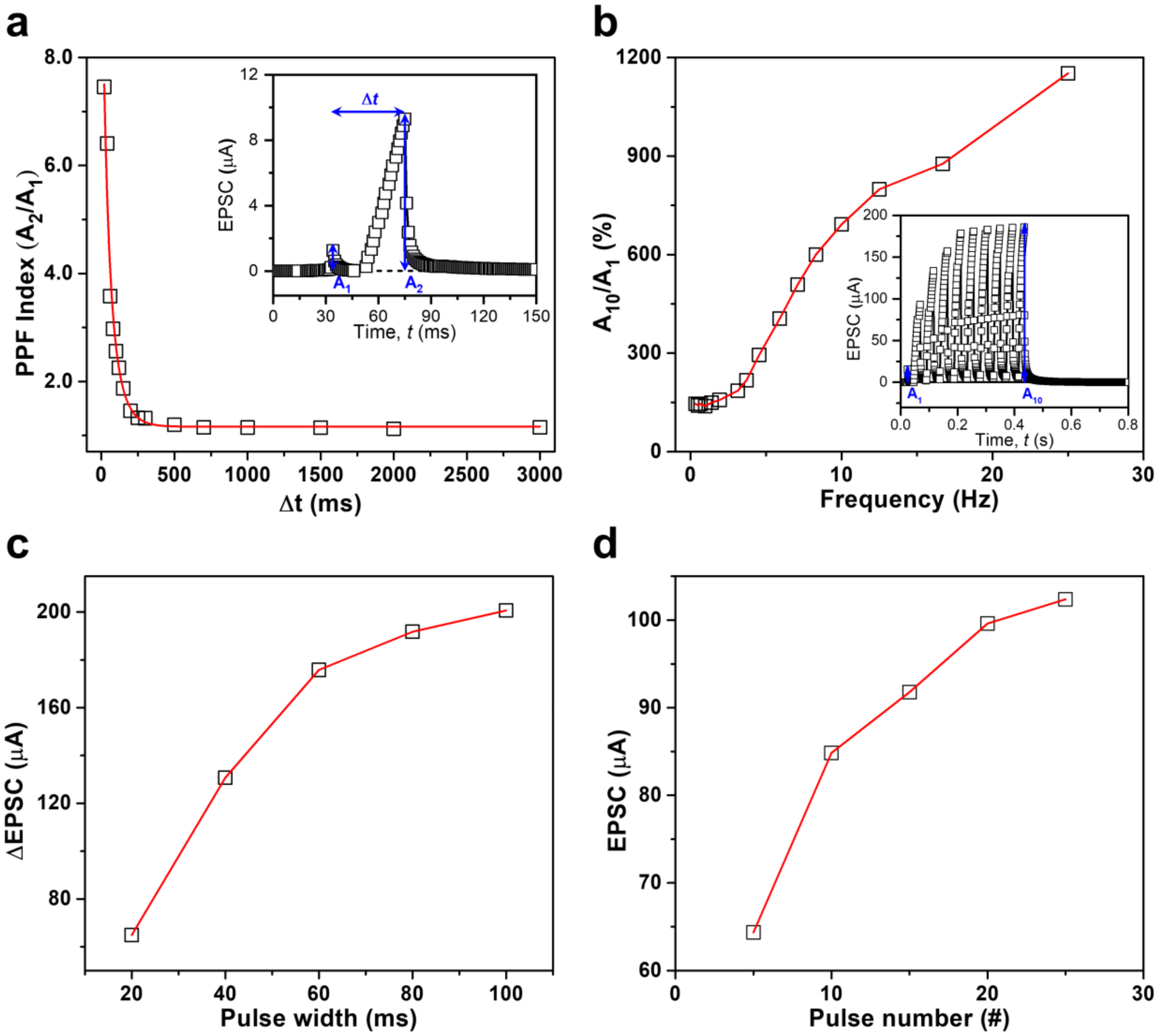

Figure S12. Robustness of PDPP3T-HD synaptic devices with the function of a) The PPF index is plotted as a function of time for an interval of two pulses. Inset: EPSC change with an interval of 20 ms. b) The current gain defined by A_{10}/A_1 is plotted as a function of frequency, indicating high-pass filtering. Inset: EPSC response to 10 pulses of 25 Hz (-4 V, 20 ms). EPSC value at 10th pulse by 10 consecutive pulses with different c) pulse width and d) pulse number.

To determine the robustness of the PDPP3T-HD-based synaptic device, we conducted several electrical measurements. Figure S9a shows that the PPF index decreases as the pulse interval time (Δt) increases from 20ms to 3000ms. Figure S9b shows that the A_{10}/A_1 percent increases with increasing frequency, indicating the presence of high-pass filtering. Figure S11c and S11d show that the EPSC varies with changes in pulse width and pulse number, respectively.

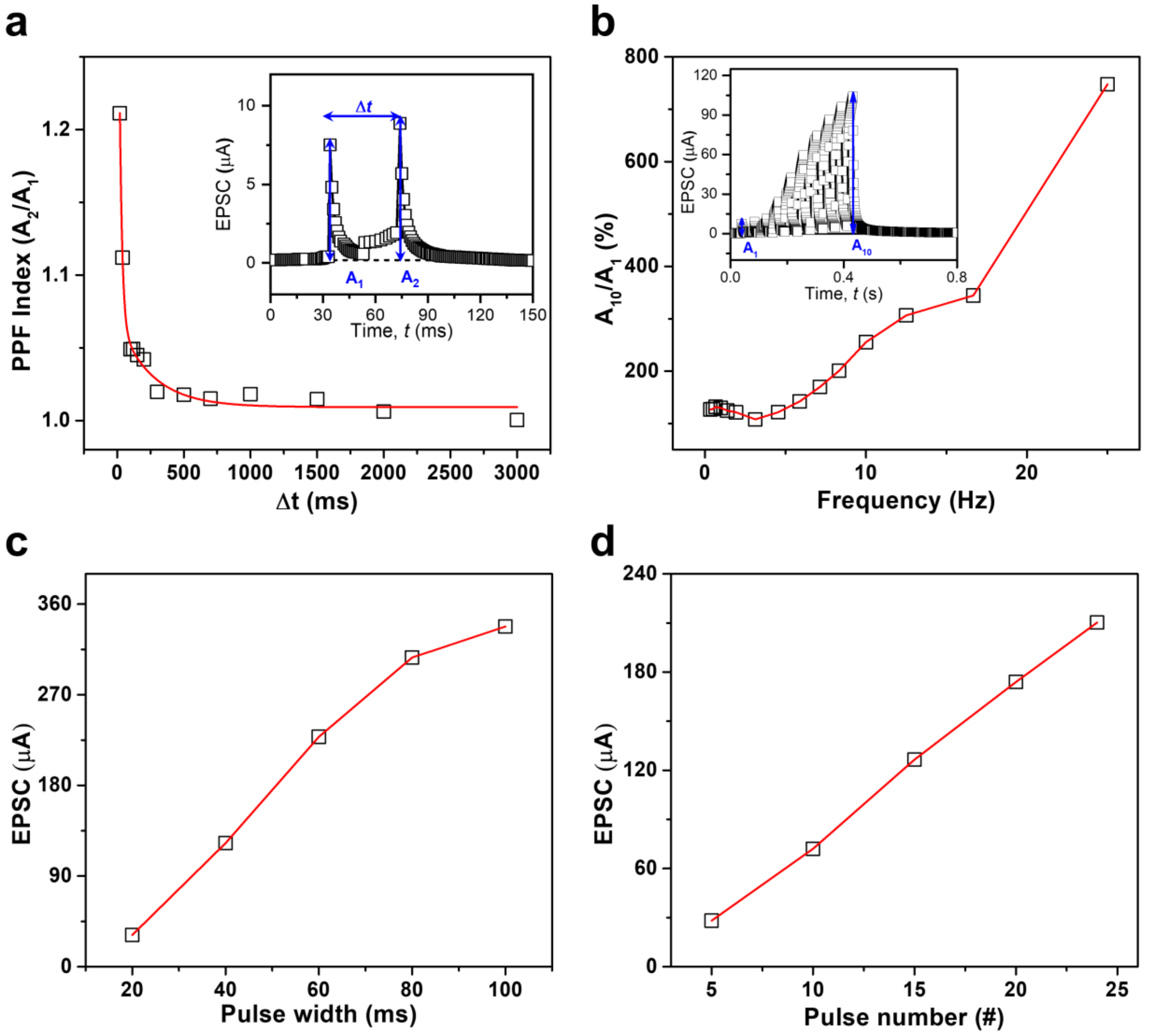

Figure S13. Robustness of PDPP3T-DT synaptic devices. a) The PPF index, defined as the ratio of A_2/A_1, is plotted as a function of the gap difference (Δt) between a series of two pulses (-4 V, 20 ms). Inset: Conductance change with an interval of 20 ms. b) The gain, defined as A_{10}/A_1, is plotted as a function of frequency from 5 to 25 Hz to characterize high-pass filtering. Inset: EPSC change to 10 pulses of -4 V, 20 ms, with a frequency of 25 Hz. EPSC responses when applying a series of 10 pulses, c) with increasing pulse width from 20 to 150 ms, and d) with increasing number of pulses from 4 to 20.

Figure S12 shows the additional synaptic performance of the PDPP3T-DT device. Figure S12a shows that the PPF index decreases as the time interval increases. Figure S12b shows the high-pass filtering properties with increasing gain with increasing frequency. Figure S12c and S12d show that the EPSC increases with increasing width and number of pulses.

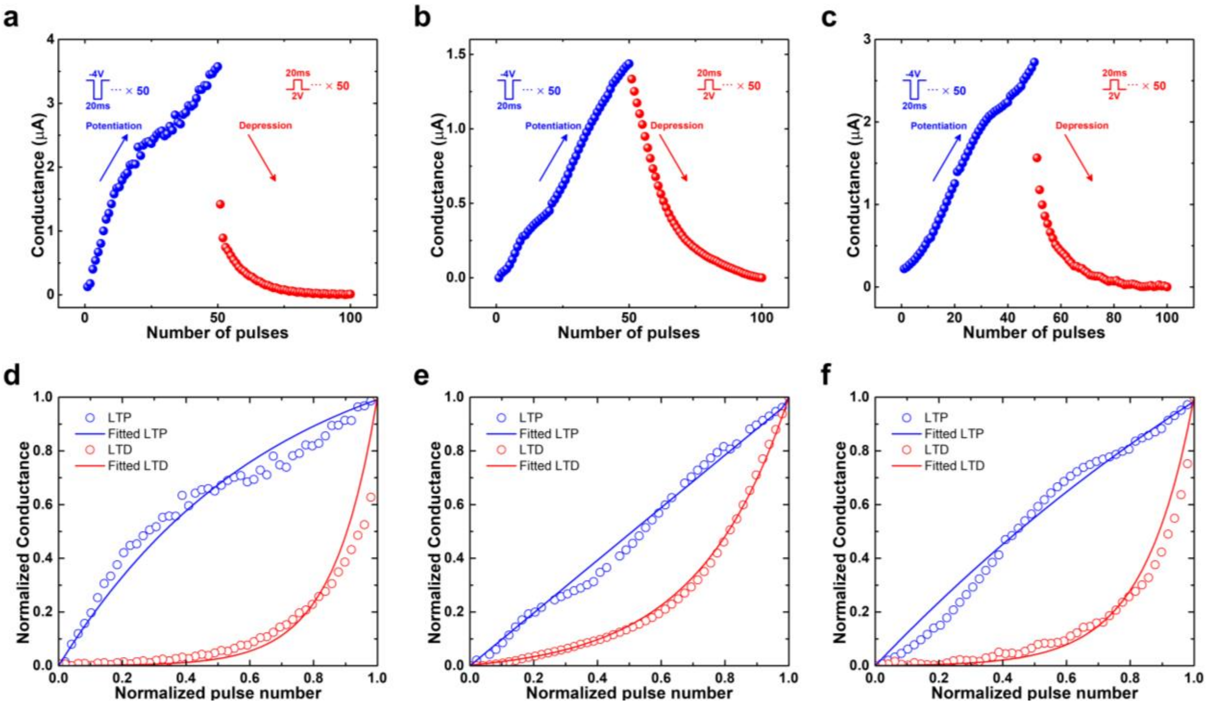

Figure S14. LTP and LTD characteristics of a) PDPP3T-HD, b) PDPP3T-OD, c) PDPP3T-DT under 100 consecutive potentiation pulses and depression pulses for 2.5 Hz. The width is fixed to 20 ms. Normalized synaptic weight change characteristics as function of electrical pulses number of d) PDPP3T-HD, e) PDPP3T-OD, f) PDPP3T-DT. Blue(red) dots and lines indicated the updated conductance for each potentiated(depressed) pulse and fitted lines, respectively.

Figure S13a–c shows the LTP/LTD characteristics of the synaptic device fabricated based on PDPP3T-HD/OD/DT after potentiation with -4 V pulses (20 ms) and depression with 2 V pulses (20 ms) with 2.5 Hz. Figure S13d–f is a curve fitted by normalizing the LTP/D data obtained through the experiment. This allows us to obtain the nonlinearity value for each device.

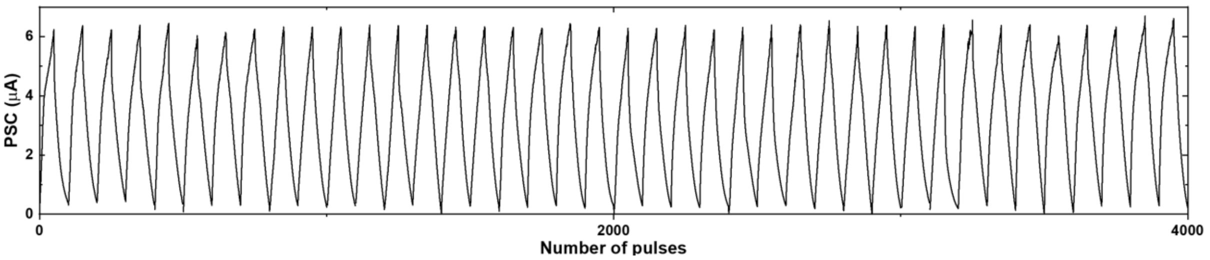

Figure S15. PSC response of a PDPP3T-OD synaptic device during 40 cycles of LTP/D with 100 consecutive pulses.

To investigate the stability of our fabricated PDPP3T-OD devices, we observed the changing PSC values as a function of the application of 40 consecutive 100 pulses. This confirmed that the device was stable even after 4000 pulses had been applied.

Supporting Information References

[1] J. P. Perdew, K. Burke, M. Ernzerhof, *Phys Rev Lett* **1996**, *77*, 3865.

[2] P. E. Blöchl, *Phys Rev B* **1994**, *50*, 17953.

[3] N. M. Alves, J. F. Mano, E. Balaguer, J. M. Meseguer Dueñas, J. L. Gómez Ribelles, *Polymer (Guildf)* **2002**, *43*, 4111.
